# Pomegranate Oil’s Potential as an Anti-Obesity Ingredient

**DOI:** 10.3390/molecules27154958

**Published:** 2022-08-04

**Authors:** Manuela Machado, Eduardo M. Costa, Sara Silva, Luís M. Rodriguez-Alcalá, Ana M. Gomes, Manuela Pintado

**Affiliations:** CBQF Centro de Biotecnologia e Química Fina-Laboratório Associado, Escola Superior de Biotecnologia, Universidade Católica Portuguesa, Rua Diogo Botelho 1327, 4169-005 Porto, Portugal

**Keywords:** conjugated linolenic acids, lipid metabolism, nutraceuticals

## Abstract

In recent years, pomegranate oil has obtained more attention due to its content of conjugated linolenic acids and possible application in the prevention of many diseases. The purpose of this work was to evaluate the potential ability of pomegranate oil to modulate obesity-related metabolism and immune response using in vitro models. In this regard, pomegranate oil was characterized in terms of fatty acids profile, tocopherols and phytosterols, and antioxidant capacity. After evaluation of the safety profile, pomegranate oil’s capacity to modulate obesity-related metabolism was evaluated through adipolysis and adipokines secretion quantification in 3T3-L1 differentiated adipocytes and hepatic lipid accumulation assay in Hep G2 hepatocytes. The immunomodulatory activity was evaluated in Caco-2 cells by quantification of pro-inflammatory cytokines IL-6, IL-8, and TNF-α. This oil showed high antioxidant capacity and was mainly composed of conjugated fatty acid, namely punicic acid. Its chemical composition was responsible for its capacity to reduce the lipid accumulation in Hep G2 cells and 3T3-L1 differentiated adipocytes. In short, pomegranate oil shows great potential for the development of functional foods and nutraceuticals targeting obesity.

## 1. Introduction

Worldwide, obesity’s prevalence has been rising. From a simplistic standpoint it results from an imbalance between energy intake and expenditure that results in an excessive expansion of adipose tissue and a set of subsequent metabolic alterations. These include low-grade inflammation, characterized by higher levels of circulating pro-inflammatory cytokines and fatty acids, which can interfere with normal insulin function and thereby induce insulin resistance, which have been implicated in b-cell dysfunction [1,2]. Indeed, obesity is the main factor responsible for metabolic syndrome, contributing to the increased risk of developing type 2 diabetes and cardiovascular diseases [1,3,4]. The current strategies, identified as more effective, to prevent and treat obesity and metabolic syndrome encompass an array of multidisciplinary approaches that range from nutritional control and exercise to psychological and pharmacological interventions. Regardless, these treatments have also been proven to be ineffective in the long run. For this reason, functional foods and nutraceuticals have emerged to complement the classic therapeutic approaches [1,5]. Many of these new options are based on bioactive compounds extracted from industrial by-products such as onion peels, citrus peels, grape and olive pomace, pomegranate seeds, flowers, and peels [1]. These by-products are especially rich in phenolic compounds, dietary fiber, and fatty acids that have been linked with an array of potential beneficial modulatory effects [1]. 

Pomegranate by-products represent up to 50% of the total fruit weight and can be a sustainable source of phenolic compounds, carotenoids, and conjugated fatty acids [6]. Pomegranate oil (PO), obtained from pomegranate seeds by green processes (cold press extraction and supercritical fluids) with little environmental impact, is mainly composed of punicic acid but is also a source of many important other classes of bioactive compounds such as tocopherols and phytosterols [7,8,9]. Their chemical composition hints at important potential obesity-related health benefits such as modulation of lipid metabolism by the reduction of lipid accumulation and lipid droplet size in adipocytes, lowering the expression of pro-inflammatory cytokines and peripheral insulin resistance, and the anti-adipogenic effect [9,10,11,12,13,14,15,16,17]. This potential is sustained by human and animal studies using a small dose of this bioactive oil. A clinical trial with 52 obese patients demonstrated a reduction in inflammatory biomarkers with a daily intake of 1g of PO [12]. Other beneficial effects such as reduction of lipid accumulation and improvement of peripheral insulin-sensitive were observed in animal studies using 1% of PO in mice diets [18,19]. Despite evidence from animal and human studies, there is a lack of mechanistic analysis to best understand the possible dose-effect relations.

Considering this, the purpose of this work was to evaluate the biological potential of organic pomegranate oil in terms of obesity-related metabolism and inflammatory response modulation supported by a detailed composition of key related bioactives.

## 2. Results

### 2.1. Chemical Composition and Antioxidant Activity

Pomegranate oil was rich in PUFAS (1017.57 ± 2.09 mg/g), namely punicic acid (the main fatty acid corresponding to 70%), oleic (7%), and linoleic acid (6.8%) (Table 1). The fatty acid profile of PO entailed an important nutritional value with low AI and TI indexes and a high HH ratio. Tocopherols and sterols appeared as another important class of lipophilic compounds in this bioactive oil. The γ-tocopherol was the major tocopherol (0.136 ± 0.02 mg/mL), and β-sitosterol was the sterol present in higher quantity (2.68 ± 0.06 mg/mL) (Table 2). This composition conferred the sample a valuable antioxidant capacity as in the L-ORAC_FL_ assay an activity of 583.04 ± 37.28 µmol Trolox equivalent/mg of oil was registered. 

### 2.2. Safety Profile: Genotoxicity and Cytotoxicity

**Figure 1:**
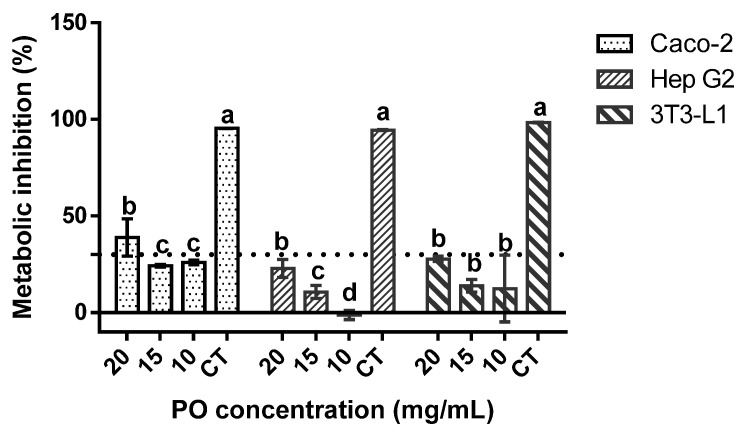
Pomegranate oil cytotoxicity towards the target cells lines at 20, 15, and 10 mg/mL. CT is the negative control (40% of DMSO). The dotted line represents the 30% cytotoxicity limit as defined by the (International Standard Organization (ISO), 2009). Different letters mean significant differences as determined by the one-way ANOVA test (*p* < 0.05).

When regarding the genotoxic profile of the sample (Table 3), no deleterious effects were found, as none of the tested concentrations of PO exerted any genotoxic effects, with and without metabolic activation, against the tested strains. When regarding the cytotoxicity results against Caco-2, Hep G2, and 3T3-L1 cells (Figure 1), none of the samples exerted any inhibitory effect upon the cellular metabolism of 3T3-L1 and Hep G2 cells, while a slight inhibitory effect was observed in Caco-2 cells at 20 mg/mL. Moreover, in Hep G2, the lowest tested concentrations seemed to promote cellular metabolism.

### 2.3. Impact on Adipolysis

**Figure 2:**
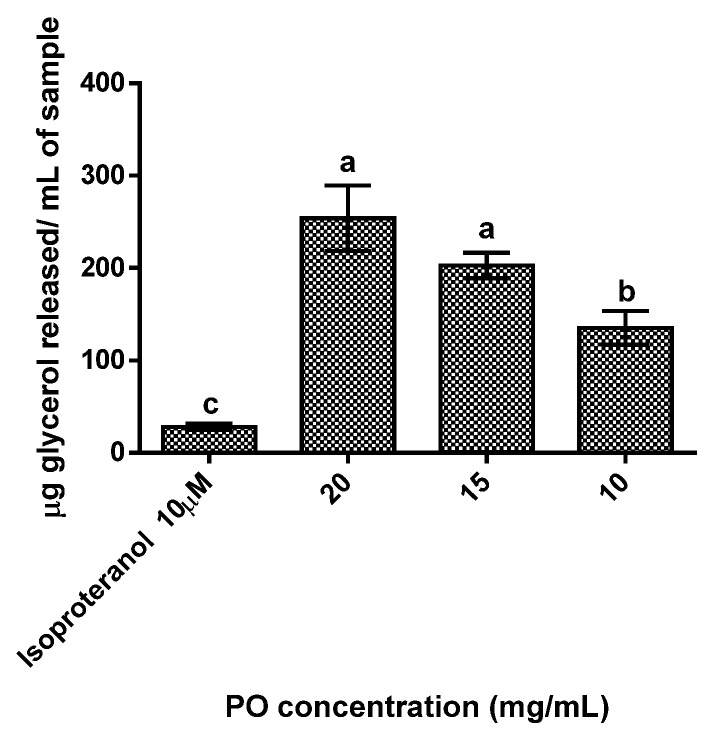
Adipolysis results for the different pomegranate oil concentrations tested. Isoproteranol at 10 µM was used as a positive control. Different letters represent significant differences as determined by the one-way ANOVA test (*p* < 0.05).

The results obtained showed a dose-dependent response (Figure 2), as the increase in PO concentration led to increases glycerol release. All tested concentration cells treated with PO showed statistically significant (*p* < 0.05) higher concentrations of glycerol released (range between 135 to 253 µg/mL) than the control (28 µg/mL), with the highest concentration of PO (20 mg/mL) exhibiting a release of glycerol 9 times higher than the control. 

### 2.4. Impact on Adipokines Secretion

**Figure 3:**
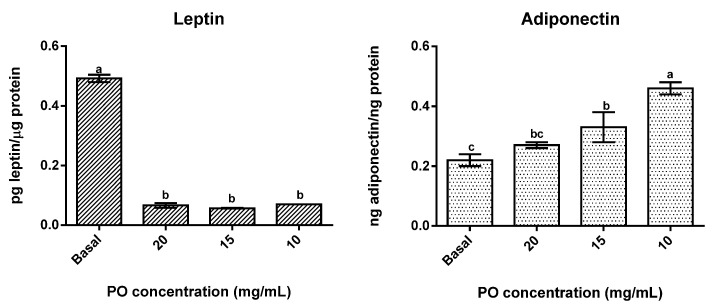
Effect of different concentrations of pomegranate oil on adipokines secretion in 3T3-L1 cells. Different letters represent significant differences as determined by the one-way ANOVA test (*p* < 0.05).

The presence of PO significantly (*p* < 0.05) reduced leptin secretion in differentiated adipocytes (Figure 3) in all tested concentrations, with no significant differences (*p* > 0.05) between concentrations being found, as all concentrations promoted reductions of around 88% in leptin secretion. On the contrary, adiponectin secretion was stimulated by the presence of PO, with the lowest concentrations tested (10 and 15 mg/mL) presenting significantly (*p* < 0.05) higher values than the control and 10 mg/mL in particular exhibiting the highest values of all. Furthermore, in this case, an inverse dose-response was observed, which means that higher concentrations of PO promoted lower secretions of adiponectin. 

### 2.5. Effect on Hepatic Lipid Accumulation

**Figure 4:**
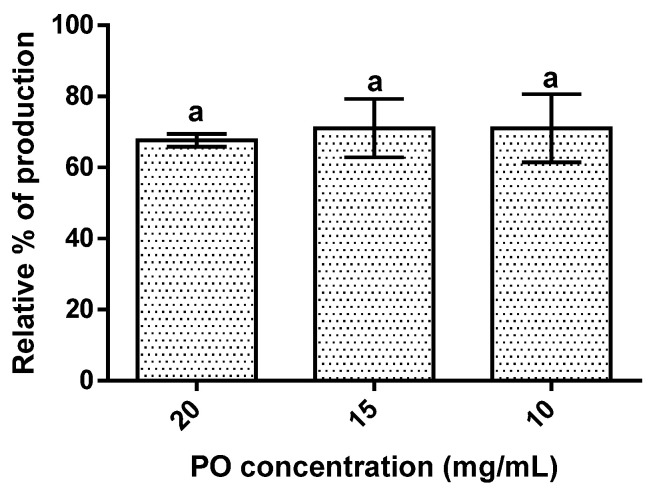
Hepatic lipid accumulation results for the different concentrations of pomegranate oil. Different letters represent significant differences as determined by the one-way ANOVA test (*p* < 0.05).

The results obtained showed that PO’s presence led to a reduction in hepatic lipid accumulation of around 30% (Figure 4). The effect of PO was independent of its concentration since an increase in PO concentration did not significantly (*p* > 0.05) change the lipidic accumulation observed. 

### 2.6. Modulation of Inflammatory Response

**Figure 5:**
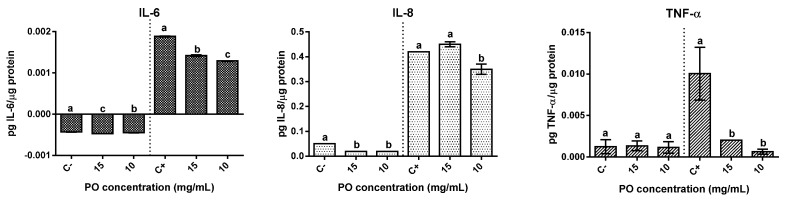
Modulation of inflammatory response in Caco-2 cells by pomegranate oil. The left part of all graphs corresponds to the non-stimulated cell’s response, and the right is related to the anti-inflammatory effect. Different letters represent significant differences as determined by the one-way ANOVA test (*p* < 0.05).

The effect on pro-inflammatory cytokines IL-6, IL-8, and TNF-α was evaluated in Caco-2 (Figure 5). In basal condition, the presence of PO resulted in differing effects. The production of IL-6 significantly (*p* < 0.05) increased in the presence of PO samples, although the overall increase was relatively low when compared with basal levels, 9% for PO at 15 mg/mL and 5% at 10 mg/mL. On the other hand, a strong significant reduction (*p* < 0.05) of IL-8 secretion was verified in relation to the basal control, with both concentrations tested exhibiting reductions above 50%. Regarding TNF-α, no significant (*p* ˃ 0.05) differences were observed when compared with the basal control. 

In cells stimulated with IL-1β, PO showed relevant anti-inflammatory effects demonstrated by significant (*p* < 0.05) reductions in the selected cytokines secretion. The presence of PO represented a significant (*p* < 0.05) reduction of IL-6 between 31% and 25% (at 10 mg/mL or 15 mg/mL, respectively) in relation to the stimulated control. In the same way, a significant (*p* < 0.05) reduction (19%) in IL-8 secretion was observed for 10 mg/mL of PO-treated cells. When regarding TNF-α a significant (*p* <0.05) reduction of around 80% and 93% in the presence of 15 and 10 mg/mL of PO, respectively, was observed. In short, the presence of PO had a significant (*p* < 0.05) positive influence upon the inflammatory response in IL-1β stimulated Caco-2 cells. 

## 3. Discussion

In the context of obesity, nutraceuticals and functional foods appeared as valuable strategies to complement the classic therapeutic and prevention solutions [20]. In this way, PO seems to be an interesting option due to its chemical composition, particularly the high amount of conjugated linolenic acids (CLNAs), and its associated potential bioactivity. When regarding the chemical composition of PO, the results presented in this work are in accordance with the ones reported by other authors [7,8,21,22]. Its punicic acid, tocopherols, and phytosterol content have been reported as being responsible for its high antioxidant capacity [23]. In this work, L-ORAC_FL_ was applied for the first time to evaluate the antioxidant potential of PO (583.04 ± 37.28 µmol Trolox equivalent/mg). However, in the available literature, the results reported were related to the DPPH assay with emphasis on the phenolic fraction, and, for this reason, a direct comparison is not possible. In addition to the chemical composition, another important factor for the use of PO as a nutraceutical or functional food ingredient is its safety. In this way, no genotoxicity effects were verified in all tested concentrations. A similar result was obtained by Meerts et al. (2009) using concentrations of PO ranging between 3 and 5000 µg/plate [24]. Furthermore, PO did not exert any inhibitory effect on 3T3-L1 and Hep G2 cells metabolism, in all tested concentrations. However, in relation to Caco-2, a slight inhibitory effect was observed at 20 mg/mL. The potential cytotoxic effects of PO were evaluated in diverse cell lines, namely 3T3-L1 and HepG2. Studies showed significant reductions in 3T3-L1 cell viability using 100 µg/mL and 25 µM of PO [25,26]. On the contrary, in HepG2 cells concentrations between 10 and 200 µmol/L did not exert any cytotoxic effect [27]. These different behaviors can be related to the differences in the chemical composition of pomegranate oil, because their compositions are dependent on the variety and the extraction methods [28,29,30,31].

Regarding the bioactive potential of PO, it showed potential to modulate obesity-related metabolism. It significantly (*p* < 0.05) inhibited the triglycerides accumulation in differentiated adipocytes, in a dose-dependent manner, through the increase in adipolysis. These results are in line with others reported by other authors whom demonstrated that punicic acid (and other conjugated linoleic acids) can increase adipolysis by the activation of peroxisome proliferator-activated receptors PPAR-γ and PPAR-α [15,32]. The agonist effect of PO in PPAR-γ can also be responsible for the reduction in adiponectin secretion observed in this work for higher PO concentrations. According to literature, adiponectin levels are reduced in the presence of high amounts of fatty acids, excess adiposity, and high lipolysis rates [33,34,35]. Moreover, the high concentration of polyunsaturated fatty acids in PO makes it more susceptible to oxidation by peroxisomes. The increase in reactive oxygen species production via peroxidation can inhibit the expression of adiponectin [36,37]. Despite this, lower concentrations of PO (10 mg/mg) were responsible for a significant (*p* < 0.05) increase in adiponectin secretion, as adiponectin has been described as functioning as an insulin-sensitizing agent (in an increase in the prevalence of insulin-sensitive adipocytes), and PO’s capacity to stimulate the production of this adipokine supports its potential use as a bioactive ingredient [3]. On the other hand, leptin secretion in adipocytes showed a significant reduction (*p* < 0.05) in the presence of PO for all tested concentrations. This is probably due to the up-regulation of PPAR-γ, as animal studies demonstrated that the activation of PPAR-γ-mediated mechanisms resulted in alteration in adiposity, reduced leptin levels and, as mentioned above, higher adiponectin levels [7,38,39,40].

Pomegranate oil was also capable of diminishing hepatic lipid accumulation. The action mechanism of this oil can be related to omega-3 fatty acids’ ability to increase hepatic fatty acid β-oxidation through the activation of PPAR-α [41,42]. In addition, polyunsaturated rich oil can also inhibit sterol-regulatory element binding protein-1C (SREBP-1c) and carbohydrate response element-binding protein (ChREBP) [41,42]. Furthermore, animal studies demonstrated that punicic acid intake (the main fatty acid in PO) reduced the accumulation of triglycerides in the liver and that lipid droplets size is also reduced [19,43].

Concerning the modulation of the immune response of Caco-2 cells, the presence of PO significantly (*p* < 0.05) reduced the secretion of IL-6 and IL-8 in non-stimulated cells as well as a significant reduction in all cytokines in in IL-1β stimulated caco-2 cells. This immunomodulatory effect can be related to the CLNAs presented in PO (punicic acid, α-eleostearic acid, and catalpic acid) as they have been considered as potential agonists of PPAR-γ and have a similar effect to those observed for conjugated linoleic acids (CLA) due to similarities in their chemical structural [44,45]. Their capacity to modulate the immune response has been associated with four different mechanisms: (i) down-regulation of eicosanoid production; (ii) increased PPAR mediated inflammatory response; (iii) suppression of inflammatory response through the regulation of the cellular transcription factor nuclear factor kappa B (NF-kB); and iv) reduction of pro-inflammatory cytokines TNF-α, IL-6 and IL-1β [46,47,48,49,50,51].

The relationship between CLNAs and CLA is important because CLNAs can be metabolized into CLA by saturation reactions. In vitro studies demonstrated that punicic acid (and other stereo- or regioisomers) can be converted into CLA (namely *cis*-9 *trans*-11 and *trans*-9 *trans*-11 isomers) by Caco-2 cells through saturation reactions catalyzed by nicotinamide adenine dinucleotide phosphate (NADP) [9]. This hypothesis was consolidated in human and animal studies, where the authors suggested that CLNAs were converted to CLA by a Δ13 saturation reaction, performed by NADPH-dependent enzyme or by an enzyme in the reductive pathway of leukotriene B4 [52,53,54,55,56]. The immunomodulatory capacity of these compounds was demonstrated by several animal and human studies that showed that the upregulation of PPRA-γ can inhibit the secretion of IL-8, IL-6, and TNF-α [12,57,58]. The individual CLNA/CLA modulation mechanism of these interleukins is not completely clear, particularly for IL-8. However, regarding the TNF-α, it is known that punicic acid exerts an anti-inflammatory effect through inhibition of TNF-α-induced priming of NADPH oxidase by targeting the p38MAPKinase/Ser345-p47phox-axis and myeloperoxidase release [59,60]. In relation to the IL-6, the reduced secretion can be explained by an inhibition of NFkB activation [61]. The capacity of PO to modulate the immune response is particularly important in the obesity context. For example, an increase in TNF-α reduces the secretion of adiponectin and promotes insulin resistance by the inhibition of the insulin receptor substrate 1 signaling pathway [62]. On the other hand, IL-6 is produced by many cell types, including immune cells and adipose tissue; their receptor is expressed in several regions of the brain, such as the hypothalamus, in which it controls appetite and energy intake, where it has a role in the regulation of energy homeostasis via suppressing lipoprotein lipase activity [63]. 

## 4. Materials and Methods

### 4.1. Chemicals and Reagents

Organic Pomegranate oil was supplied by All Organic Treasure (Wiggensbach, Germany). Tritridecanoin was obtained from Larodan (Solna, Sweden); HPLC grade methanol, hexane, dimethylformamide (DMF), ethanol, acetone, and dichloromethane were obtained from VWR (PA, USA), and sodium methoxide from Acros Organics (NJ, USA). The standards Supelco 37 and CRM-164, trolox, phosphate buffered saline (PBS), 3-(4,5-dimethylthiazol-2-yl)-2,5-diphenyltetrazolium bromide (MTT), fluorescein, 2,2′-azobis(2-methylpropionamidine) dihydrochloride (AAPH) sulfuric acid (97%), and dimethyl sulfoxide (DMSO) were obtained from Sigma (St. Louis, MO, USA). Tocopherols and phytosterol standards (α-tocopherol, Δ-tocopherol, γ-tocopherol, β-sitosterol, and cholesterol) were obtained from Extrasynthese (Lyon, France), IL-1β from Invitrogen (Waltham, MA, USA). Random methyl-β-cyclodextrin (RMBC) kindly provided by CycloLab (Budapest, Hungary). Dulbecco’s Modified Eagle’s Medium (DMEM) and non-essential amino acids (NEAA) were obtained from Gibco (Thermo Scientific, Waltham, MA, USA), Fetal Bovine Serum from (FBS; Biowest, Nuaillé, France), Penicillin-Streptomycin-Fungizone from Lonza, (Basel, Belgium), and Calf Bovine Serum (CBS), Iron Fortified from ATCC (Manassas, VA, USA). Ames FT™ Mutagenicity Test Kit was obtained from Moltox (Boone, NC, USA), adipolysis Assay Kit from Abcam (Abcam ab133115), Leptin ELISA kit (Abcam, ab199082), Mouse Adiponectin ELISA kit (Abcam, ab18785, Hepatic Lipid Accumulation Kit (Abcam ab133131) and ELISA using the Human IL-6 Elisa Kit High Sensitivity (Abcam, ab6042) were obtained from Abcam (Cambridge, UK). Legend Max Human Elisa Kit IL-8 and the Legend Max Human Elisa Kit TNF-α were obtained from BioLegend (San Diego, CA, USA) and BCA Pierce Assay Kit from Thermo Scientific.

### 4.2. Fatty Acids Profile

The determination of PO fatty acids profile was carried as previously described by Sousa et al. 2022 [64] with slight modifications. Briefly, 15 mg of oil was added to 200 µL of tritridecanoin, followed by 2.26 mL of methanol, 800 µL of hexane, and 240 µL of sodium methoxide (5.4 M). Samples were vortexed and incubated at 80 °C for 10 min. After cooling in ice, 1.25 mL of DMF and 1.25 mL of sulfuric acid (3 M) in methanol was added, the samples vortexed and incubated at 60 °C for 30 min. After cooling, samples were vortexed and centrifuged (1250× *g*; 18 °C; 5 min). The upper layer containing fatty acids methyl esters was collected and analyzed in a gas chromatograph HP6890A (Hewlett-Packard, Avondale, PA, USA), equipped with a flame ionization detector and a BPX70 capillary column (60 m × 0.25 mm × 0.25 μm; SGE Europe Ltd., Courtaboeuf, France). Analysis conditions were as follows: injector (split 25:1; injection volume 1 µL), injector and detector temperatures were 250 °C and 275 °C, respectively; hydrogen was used as a carrier gas at a flow rate of 1 mL/min. The oven temperature was initially at 60 °C and then increased to a final temperature of 225 °C. Supelco 37 and CRM-164 were used for the identification of fatty acids. 

Furthermore, atherogenic index (AI), thrombogenic index (TI), and hypocholesterolemic/hypercholesterolemic ratio (HH), concerning lipid quality were calculated according to Alba et al. 2019 [65] using the following equations:

(1) $$AI=\frac{\left[C12:0+4\times \left(C14:0\right)+C16:0\right]}{\left(\sum MUFA+\sum PUFA\;n6+\sum PUFA\;n3\right)}$$

(2) $$TI=\frac{\left(C14:0+C16:0+C18:0\right)}{\left[0.5\times \sum MUFA+0.5\times \sum PUFA\;n6+3\times \sum PUFA\;n3+\left(\frac{\sum PUFA\;n3}{\sum PUFA\;n6}\right)\right]}$$

(3) $$HH=\frac{\left(C18:1n9+C18:2n6+C18:3n3+C20:4n6+C20:5n3\right)}{\left(C14:0+C16:0\right)}$$

### 4.3. Tocopherols and Phytosterols

Tocopherols and phytosterols were quantified according to the method proposed by Pokkanta et al. 2019 [66] with slight modifications. Oils were diluted 1:1 (*v*/*v*) in dichloromethane and filtered through a 0.22 µL syringe filter. A sample volume of 5.0 μL was used for the chromatographic analysis. Quantitative analysis of phytosterols was operated using an HPLC system consisting of an Agilent HPLC 1260 connected to a diode array detector (Model 1260 DAD WR, Agilent Technologies, Palo Alto). Tocopherols were analyzed using an HPLC system consisting of a Beckham 126 and a fluorescence detector (Varian detector). The separation column was ACE equivalence 5, C18, 250 × 46 mm (VWR, USA), the mobile phase was methanol in an isocratic elution, the flow rate was 1.0 mL/min, and the column was kept at constant temperature (30 °C). Tocopherols were detected by fluorescence (excitation at 294 nm and emission at 326 nm) and phytosterols were detected by absorbance at 210 nm. Calibration curves (2.5–0.00025 mg/mL) of pure standards of α-tocopherol (RT: 14.93 min), γ-tocopherol (13.69 min) for tocopherols, and concentrations between (10–0.0625 mg/mL) of β-sitosterol (RT: 21.22 min), and cholesterol (RT: 18.19 min) were used for quantification.

### 4.4. Antioxidant Capacity

Pomegranate oil’s antioxidant capacity was analyzed with lipophilic ORAC (L-ORAC_FL_) through an adaption of the procedure first described by Poyato et al. 2013 [67]. Briefly, 10 µL of oil was suspended in 400 µL of an ethanol:acetone (7:3, *v/v*) solution and added to 4.6 mL of a 7% (*w/v*) RMCD solution (1:1, acetone:water, *v/v*). The mixture was then shaken at 800 rpm in an orbital shaker for 1 h at room temperature. For standards preparation, a Trolox stock solution (1 mM) was prepared in PBS (75 mM, pH 7.4). From here, a Trolox work solution (100 µM) was prepared through dissolution with RMCD, and further diluted with PBS to obtain standards with concentrations ranging from 10 to 80 µM. RMCD solution was also used to dilute samples and as blank. 

The L-ORAC_FL_ assays were performed in 96-well U bottom, polypropylene, black microplates (Thermo ScientificTM, NuncTM, Denmark), with 20 µL of the sample (standards or blank) being added to 120 µL of fluorescein (116.66 nM), and equilibrated for 10 min at 37 °C. Afterwards, the reaction was initiated through the addition of 60 µL of AAPH (48 mM), and immediately placed in a microplate reader (Synergy H1, Biotek Instruments, Winooski, VT, USA) with fluorescence being read (485 nm excitation and 580 nm emission), throughout 80 min in 1 min intervals. Results were expressed as µmol Trolox equivalent/mg.

### 4.5. Genotoxicity Evaluation—AMES Assay

AMES assay was executed in accordance with the OECD guideline 471 [68] using the Ames FT™ Mutagenicity Test Kit according to the manufacturer’s instructions. Briefly, *Salmonella typhimurium* TA98 and TA100 strains were grown overnight, at 37 °C, after which they were placed in an orbital incubator until an OD_650_ between 1.0 and 1.4 was obtained. Samples were tested at 20, 15, 10, and 5 mg/mL. Two controls were assessed: one with chemical control capable of inducing bacterial mutation and another with water instead of test sample as solvent control. Assays were performed with and without metabolic activation. After 90 min of exposure at 37 °C, in exposure media and under constant stirring, reversion media was added and samples were transferred to a 384-well plate. Plates were then incubated for 48 h at 37 °C after which the number of wells that changed from purple to yellow were counted. According to the kit instructions a positive result (genotoxic effect) equaled an increase of ≥2-fold in a treatment group in relation to the corresponding solvent control.

### 4.6. Cell Lines Growth Conditions

Human Caucasian colon carcinoma epithelial cells, Caco-2 (ECACC 86010202), were obtained from the European Collection of Authenticated Cell Cultures. Human hepatocellular carcinoma cells, Hep G2 (ATCC HB-8065), and mouse pre-adipocytes 3T3-L1 (ATCC CL-173) were acquired from American Type Culture Collection. Caco-2 and Hep G2 cells were cultured using DMEM (4.5 g/L glucose, L-glutamine without pyruvate) (supplemented with 10% (*v/v*) of FBS and 1% (*v/v*) of Penicillin-Streptomycin-Fungizone. Caco-2 cells’ media were also supplemented with 1% (*v/v*) NEAA. Pre-adipocytes were cultured in DMEM with 10% (*v/v*) of CBS, Iron Fortified and 1% (*v/v*) of Penicillin-Streptomycin-Fungizone. All incubations were carried out 37 °C in a humidified atmosphere of 95% air and 5% CO_2_.

### 4.7. Cytotoxicity

The cytotoxicity assay was performed according to ISO 10993-5 [69], with slight modifications, using the MTT viable dye. Briefly, cells were seeded at 1.0 × 10^5^ cells/mL into wells of 96-well tissue culture plates (Thermo Scientific, Copenhagen, Denmark) and allowed to adhere for 24 h. Afterwards, the media was replaced by 100 µL of PO diluted in culture media at various concentrations (5–25 mg/mL). Media with DMSO (40%) was used as negative control and fresh medium as a positive control (cells in normal growth conditions). After 24 h of exposure, 100 µL of MTT (solution (0.5 mg/mL) was added to each well and the plates were incubated at 37 °C, in the dark. After 2 h, MTT solution was removed and 100 µL of DMSO were added. The plates were shaken protected from light for 10 min and absorbance was read at 570 nm using a microplate reader (Synergy H1, Biotek Instruments, Winooski, VT, USA).

### 4.8. Adipolysis

The PO effect upon 3T3-L1 cells adipolysis was performed using the Adipolysis Assay Kit according to the manufacturer’s instructions. Briefly, cells were seeded at 3.0 × 10^4^ cells/mL in 96-well tissue culture plates (Thermo Scientific, Copenhagen, Denmark) and allowed to grow until confluence. Two-day post confluence the media were replaced by differentiation induction media provided in the kit. Following induction, the media were replaced every three days by insulin media until more than 80% of the cells were differentiated. Afterwards, the medium was replaced by PO containing medium at various concentrations (10–20 mg/mL) and incubated for 24 h. Isoproterenol solution (10 µM) was used as a positive control and plain medium as a negative control. The glycerol concentration was measured by adding a glycerol-free reagent to 25 µL of cell supernatants, incubating the mixture for 15 min at room temperature, and measuring the absorbance (540 nm) in a microplate reader (Synergy H1, Biotek Instruments, Winooski, VT, USA).

### 4.9. Adipokines Secretion

Pre-adipocytes (3T3-L1 cells) were seeded at 2.0 × 10^4^ cells/mL and differentiated until more than 80% of the cells were differentiated. Afterwards, the medium was replaced by a medium with PO (10–20 mg/mL) and incubated for 24 h. For basal activity plain media were used as control. At the end of the assay, supernatants were collected, centrifuged to remove debris, and stored at −80 °C until further analysis. Adiponectin and leptin detection was performed by enzyme-linked immunosorbent assay (ELISA) using the Mouse Leptin ELISA kit and Mouse Adiponectin ELISA kit according to the manufacturer’s instructions. The protein content of samples was determined using the BCA Pierce Assay Kit. Leptin values were obtained in pg/µg of protein in the sample and adiponectin in ng/ng of protein. 

### 4.10. Hepatic Lipid Accumulation

Hepatic lipid accumulation assay was performed using Hepatic Lipid Accumulation Kit according to the manufacturer’s instructions. Briefly, hepatocytes, seeded at 10^4^ cells/mL in a 96-well plate, were allowed to adhere. After 24 h the media were replaced by fresh media with PO (10–20 mg/mL) or chloroquine (25 μM steatosis induction control). After 72 h of exposure, cells were stained with Oil Red O and the absorbance at 490 nm was measured using a microplate reader (Synergy H1, Biotek Instruments, Winooski, VT, USA). The results were expressed in relative percentage of control production. 

### 4.11. Caco-2 Immunomodulation

Caco-2 cells were seeded at 2.5 × 10^5^ cells/well in 24-well microplates and incubated for 24 h. After 24 h, the culture media was carefully replaced with media supplemented with PO at 15 and 10 mg/mL, and the plate was re-incubated for another 24 h. As an inflammation control, IL-1β was used at 10 ng/mL while for basal activity control plain media were used. At the end of the assay, supernatants were collected, centrifuged to remove debris, and stored at −80 °C for further analysis. 

Interleukins 6 (IL-6) and 8 (IL-8) and Tumor Necrosis Factor-alpha (TNF-α) detection were performed by ELISA using the Human IL-6 Elisa Kit High Sensitivity, the Legend Max Human Elisa Kit IL-8 and the Legend Max Human Elisa Kit TNF-αaccording to the manufacturer’s instructions. Protein content of samples was determined using the BCA Pierce Assay Kit. Interleukin values were obtained in pg/ng of protein in the sample. 

### 4.12. Statistical Analysis

Minitab 17 (Minitab, LCC, State College, PA, USA) was used to carry out statistical analysis. All data was reported as a mean ± standard deviation. Shapiro-Wilk’s test was used to confirm the normality of data distribution. The results obtained were tested at a 0.05 significance level using a one-way analysis of variance (ANOVA), followed by a multiple comparisons test (Tukey) to identify statistically significant differences.

## 5. Conclusions

Pomegranate oil can be a valuable ingredient for functional foods or nutraceuticals due to its high content in bioactive molecules, particularly CLNAs. This bioactive oil plays an important role in modulating obesity-related metabolism through the reduction in lipid accumulation. The reduction in lipid accumulation combined with the increase in adiponectin can provide a benefit to the management of insulin resistance. Moreover, their capacity to modulate the immune response makes PO a great source of bioactive molecules to ameliorate a metabolic syndrome clinical condition. 

## Figures and Tables

**Table 1:** Fatty acids profile of pomegranate oil. All values are expressed in mg/mL (mean ± standard deviation).

Fatty Acids	
C16	38.80 ± 12.32
C18	35.19 ± 10.80
C18:1 *t*9	2.53 ± 0.62
C18:1 *t*10	4.30 ± 0.64
C18:1 *t*11	5.14 ± 1.39
C18:1 *t*12	4.00 ± 0.93
C18:1	96.08 ± 3.75
C18:1 *c*11	6.72 ± 2.10
C18:1 *c*12	1.36 ± 0.32
C18:1 *c*13	1.20 ± 0.33
C18:2 *t*9*t*12	1.37 ± 0.41
C18:1 *c*4*/t*15	5.04 ± 1.40
C18:2	90.76 ± 3.27
C18:3 *c*6*c*9*c*13	1.24 ± 0.42
C18:3 *c*9*c*12*c*15	10.38 ± 0.60
C20	5.83 ± 1.61
C18:2 *c*9*t*11	6.59 ± 1.84
C20:1	12.00 ± 3.32
C20:4	2.11 ± 0.67
C18:3 *c*9*t*11*c*13	858.16 ± 11.03
C18:3 *t*9*t*11*t*13	10.67 ± 0.66
C18:3 *t*9*t*11*c*13	36.29 ± 5.27
C24	1.51 ± 0.15
∑Fatty acids	1235.77 ± 41.67
∑SFA	81.33 ± 24.90
∑MUFA	138.37 ± 14.82
∑PUFAS	1017.57 ± 2.09
∑PUFAS n3	916.74 ± 4.09
∑PUFAS n6	100.84 ± 6.19
**Nutritional Quality Indexes**	
AI	0.03 ± 0.01
TI	0.03 ± 0.00
HH	3.13 ± 0.78

C16—palmitic acid; C18—stearic acid; C18:1 t9—elaidic acid, C18:1 t10, C18:1 t11, C18:1 t12—*trans* isomers of oleic acid C18:1 *c*9—oleic acid; C18:1 *c*11—*cis*-vaccenic acid; C18:1 c12, C18:1 c13—cis isomers of oleic acid; C18:2 *c*9*c*12—linoleic acid; C18:3 c6c9c13—γ-Linolenic acid; C18:3 *c*9*c*12*c*15—linolenic acid; C20—Arachidic acid; C18:2 c9t11—conjugated linoleic acid; C20:1—Eicosenoic acid; C20:4—Arachidonic acid; C18:3 c9t11c13—punicic acid; C18:3 t9t11t13—C24—Lignoceric acid, SFA—saturated fatty acids, MUFA—monounsaturated fatty acids, PUFA—polyunsaturated fatty acids, AI—atherogenic index; TI—thrombogenic index); HH—hypocholesterolemic/hypercholesterolemic ratio.

**Table 2:** Phytochemical profile of pomegranate oil. All values expressed in mg/mL (mean ± standard deviation).

Tocopherols	
Δ-tocopherol	0.088 ± 0.01
γ-tocopherol	0.136 ± 0.02
**Sterols**	
Cholesterol	1.10 ± 0.05
β-sitosterol	2.68 ± 0.06

**Table 3:** Results obtained for PO samples AMES genotoxicity assay against *S. typhymurium* TA98 and TA100. All values represent the average number of positive (revertant) wells ± standard deviation.

	TA98		TA100	
	Without S9	With S9	Without S9	With S9
Solvent control (Baseline)	1.33 ± 0.58	1.67 ± 0.58	8.33 ± 0.58	8.67 ± 2.08
Positive Control	46.67 ± 0.58	47.33 ± 0.58	19.67 ± 2.31	47.67 ± 0.58
20 mg/mL	1.33 ± 0.58	0.33 ± 0.58	5.33 ± 2.52	9.00 ± 4.36
15 mg/mL	1.67 ± 0.58	0.67 ± 1.15	8.00 ± 2.00	7.00 ± 1.00
10 mg/mL	0.33 ± 0.58	0.67 ± 0.58	7.00 ± 1.73	6.67 ± 1.53
5 mg/mL	0.00 ± 0.00	0.67 ± 0.58	6.33 ± 2.89	6.67 ± 3.09

## Data Availability

The data presented in this study are available on request from the corresponding author. The data are not publicly available due to confidentiality.
